# German translation and validation of the Pelvic Organ Prolapse/Incontinence Sexual Questionnaire–IUGA revised (PISQ-IR)

**DOI:** 10.1007/s00192-016-2969-9

**Published:** 2016-02-16

**Authors:** Gerda Trutnovsky, Eva Nagele, Daniela Ulrich, Thomas Aigmüller, Daniela Dörfler, Ingrid Geiss, Evi Reinstadler, Johannes Angleitner-Flotzinger, Jean-Jacques Ries, Vesna Bjelic-Radisic, on behalf of the Austrian Urogynecology Working Group

**Affiliations:** Department of Gynecology, Medical University of Graz, Auenbruggerplatz 14, 8036 Graz, Austria; Department of Gynecology, Vienna General Hospital, Währinger Gürtel 18-20, 1090 Wien, Austria; Department of Gynecology, Hospital Lilienfeld, Im Tal 2, 3180 Lilienfeld, Austria; Department of Gynecology, Hospital Dornbirn, Lustenauerstraße 4, 6850 Dornbirn, Austria; Department of Gynecology, St.Vincents Hospital Ried, Schlossberg 1, 4910 Ried im Innkreis, Austria; Department of Gynecology, Hospital Aarau, Tellstrasse, 5001 Aarau, Switzerland

**Keywords:** PISQ-IR, Sexual function, Pelvic floor disorder, Questionnaire, Validation

## Abstract

**Introduction and hypothesis:**

Condition-specific sexual questionnaires are essential for clinical trials and important patient-reported outcome measures. The aim of the study was to translate the Pelvic Organ Prolapse/Incontinence Sexual Questionnaire–International Urogynecology Association Revised (PISQ-IR) into German and to clinically validate it in a German-speaking population.

**Methods:**

The translated PISQ-IR was linguistically validated in two rounds of cognitive interviews. The final instrument was psychometrically validated in women presenting to urogynecological clinics with pelvic floor dysfunction. For analysis of criterion validity, three related self-reported measures were administered: the Female Sexual Function Index (FSFI), the Kings Health Questionnaire (KHQ), and the 36-Item Short Form Health Survey (SF-36). For external validity, PISQ-IR subscales were compared to the clinical-measures Pelvic Organ Prolapse Quantification system (POP-Q) stage, pelvic floor muscle tone, and Oxford Grading Scale. Descriptive statistics, floor and ceiling effects, internal consistency using Cronbach’s alpha coefficient, and Pearson correlations were calculated for all PISQ-IR subscales.

**Results:**

The PISQ-IR was completed by 197 women, out of whom 66 (33.5 %) considered themselves not sexually active (NSA) and 131 (66.5 %) as sexually active (SA). Participants’ mean age was 57 ± 12 years; 50 % were diagnosed with symptomatic POP, 74 % with urinary incontinence (UI) and 4 % with anal incontinence (AI). The PISQ-IR subscales were analyzed separately for SA and NSA women with Cronbach’s alpha coefficients ranging from 0.64 to 0.94. Moderate to high correlations were observed between PISQ-IR subscales and related quality of life (QoL) scales and corresponding FSFI scales.

**Conclusion:**

Initial testing of the German PISQ-IR suggests it is an internally consistent and valid tool for use in clinical practice and research.

## Introduction

Sexual health is an integral part of quality of life (QoL) and may play an important role up to and into to old age [1, 2]. Pelvic floor dysfunction (PFD) increases with age and is likely to negatively affect sexual activities [3–6]. Urinary (UI) and fecal (FI) incontinence have been associated with low libido, dyspareunia, and avoidance of sexual activity for fear of losing urine or stool [3, 7–9]. Pelvic organ prolapse (POP) may cause discomfort, decrease sexual excitement, and influence a woman’s decision not to be sexually active [3, 10–12].

Evaluation of sexual activity and function should be an integral part of urogynecologic assessment, since this information is essential for therapy planning. Comprehensive physician-led interviews covering different aspects of sexuality are still rare, and questionnaires are increasingly used for evaluation of female sexual function. Condition-specific sexual questionnaires are essential for clinical trials and important patient-reported outcome measures. Thus, the International Urogynecology Association (IUGA) developed a new questionnaire for evaluating the impact of PFD on sexual function and activity: the Pelvic Organ Prolapse/Urinary Incontinence Questionnaire-IUGA revised (PISQ-IR) [5, 6]. In contrast to previous tools, this PFD-specific questionnaire addresses sexually active (SA) women as well as women without a partner and those who consider themselves not to be sexually active (NSA). The PISQ-IR was validated in >500 women seeking treatment for UI and/or anal incontinence (AI) and/or POP in several clinical sites from across the USA and UK. The questionnaire is based in a multicultural framework, and translation and validation in several languages is ongoing or already completed [13, 14].

In German there are currently three validated questionnaires on sexual function available: the Female Sexual Function Index (FSFI) [15], the Sexual Activity Questionnaire (SAQ) [16], and the McCoy Female Sexuality Questionnaire (MFSQ). However, none of them is PFD-specific, and there is an urgent need for a valid sexual function scale for use in urogynecological research. The aim of this study was to translate the PISQ-IR into German and to clinically validate it in a German-speaking population of women with PFD.

## Materials and methods

### Original PISQ-IR questionnaire

The validity, reliability, and responsiveness of the original English-language version of the PISQ-IR was established in a population of women with PFD [9]. The questionnaire consists of two sections: one for NSA and one for SA women and comprises 12 or 21 items, respectively. The division is based on the result of the core-branching item 1, where women are asked to indicate whether they consider themselves to SA or NSA, with or without a partner. The two sections are analyzed independently, each consisting of two domains and different subscales. The NSA section contains the following four subscales: condition-specific reasons for sexual inactivity (NSA-CS), partner- and personal-interest-related reasons for sexual inactivity (NSA-PR), global rating of sexual quality (NSA-GQ), and condition impact (NSA-CI). The SA section contains the following six subscales: sexual arousal and orgasm (SA-AO), partner-related issues (SA-PR), condition-specific issues (SA-CS), global quality (SA-GQ), sexual desire (SA-D), and a condition-specific subscale (SA-CS). All subscales are analyzed separately, and calculation of a summary score is not recommended [17]. In the NSA group, a higher score indicates greater negative impact of urogynecological symptoms, whereas for SA women, a higher score indicates less impact and better sexual function.

### Translation and linguistic validation

Translation into German was undertaken by a German-speaking Austrian-native translator with profound English language skills and a broad knowledge of questionnaire development and sexual issues. Special consideration was given to avoid offending or misleading phrases and ensure cultural acceptance without loosing the particular context and intent of each question.

In a subsequent community review process, the translated instrument was trialed for it is consistency and acceptance. During ten cognitive interviews with women with PFD, the intent and wording of each item was discussed. In a one-on-one session, taking between 30 and 60 min, women were probed about their initial reactions, their understanding of the content, and whether there was any alternative wording they would find easier to understand or relate to. Based on the results of the cognitive interviews, the instrument was revised and subjected to a second round of ten cognitive interviews. After finalizing the wording of the German version, each question was translated back into English by an independent Austrian-native English-speaking translator. The completed Forward-Translation/Back-Translation form was reviewed by the IUGA PISQ-R working group.

### Psychometric validation

For clinical validation, the German language version questionnaire was then administered to women presenting to urogynecology clinics. According to the PISQ-IR Translation Protocol enrolment of 220 women—120 SA and 100 NSA —was planned. Inclusion criteria were a diagnosis of UI and/or AI and/or POP and sufficient German language skills. Exclusion criteria were diagnosis of vulvodynia, painful bladder syndrome, or chronic pelvic pain. A convenience sample of women meeting these criteria was asked to participate and was enrolled in the validation study. Informed consent was collected from all participants, and ethics approval was obtained from the university and local ethics committees.

A standardized interview included current symptoms, obstetric and gynecologic history, and past and ongoing urogynecologic therapies. A physical examination was performed to stage POP according to the POP-Q classification [18]. Clinical assessment of pelvic floor muscles was performed by vaginal digital palpation for assessment of muscle tone and contractility. The six-point Oxford Grading Scale was used to rate contractility from 0 (no muscular action) to 5 (strong squeeze and lift) [19]. For analysis of criterion validity, three related and previously validated self-reported measures were administered in addition to the PISQ-IR. The FSFI is a 19-item questionnaire for assessment of several aspects of sexual functioning in a general population and contains the following six subscales: desire, arousal, lubrication, orgasm, satisfaction, and pain, with higher scores indicating better sexual function [15]. The Kings Health Questionnaire (KHQ) is a 32-item, condition-specific instrument to assess quality of life (QoL) in women with UI and contains nine subscales: general health perception, incontinence impact, role limitations, physical limitations, social limitations, personal relationship, emotional problems, sleep and energy disturbances, and symptom severity, with higher values indicating worse QoL [20]. The 36-Item Short Form Health Survey (SF-36) is generic and widely used to assess perceived health state and QoL comprising eight health domains: Physical Functioning, Emotional-role Functioning (role limitations due to emotional problems), Social Functioning, Bodily Pain, Mental Health (psychological distress and well-being), Physical-role Functioning (role limitations due to physical health), Vitality (energy and fatigue), and General Health, with higher scores indicating better QoL [21]. Participating women were handed the survey packet comprising all four questionnaires and asked to return the completed forms in a prepaid envelope via mail.

### Statistical analysis

Differences in clinical characteristics and questionnaire data between NSA and SA women were assessed with Student* t* tests. Descriptive statistics and floor and ceiling effects, i.e. percentages of patients with the lowest and highest possible scores, were calculated for all PISQ-IR subscales. Internal consistency was assessed with the use of Cronbach’s alpha coefficient. Subscales were scored when at least half of the items were answered; transformed summation, i.e. transformation of all items to a 0–100 range, was applied. According to the recommendation of the PISQ-IR study group, imputation of missing values was not performed [17]. To assess criterion validity, PISQ-IR subscales were compared with the reference questionnaires FSFI (for SA women only), KHQ (for women with diagnosed UI only), and SF-36. For external validity, PISQ-IR subscales were compared with clinical-measures POP-Q stage, Oxford Grading Scale and pelvic floor muscle tone. For analysis, Pearson correlations or Spearman’s rank-sum correlations were use, with values of 0.3–0.5 indicating weak, >0.5–0.7 indicating moderate, and >0.7 indicating strong correlation. A* p* value <0.05 was regarded as significant. Scale responsiveness and test–retest reliability were established for the original questionnaire and were not assessed in this study.

## Results

### PISQ-IR translation

During the translation process and cognitive interviews, several challenging key phrases, i.e. “bulging in the vagina”, or “sexually inferior” were identified and extensively discussed before the most appropriate wording was found. For the core-branching question about sexual activity, the phrase “sexually active with or without sexual intercourse” was added to the phrase “with or without a partner”. The number and contents of items remained unchanged, but a short introduction explaining the rationale and content of the questionnaire was added. The 20 women who participated in the cognitive interviews were all diagnosed with at least one PFD—60 % with UI and 55 % with POP–and had a mean age of 61.4 years [standard deviation (SD 8.8)]. The final German version was well received and understood. However, at the end of the validation, process a nonresponse rate of up to 55 % was observed for some items, and this seemed to be due to the differing answering formats. As a consequence, the answering format of two questions—Q4 for NSA and Q19 for SA women—was modified to fit the other answering formats (see Appendix 1).

### PISQ-IR validation

Five urogynecological centers throughout Austria and one Swiss center participated in the validation process. A total of 215 women returned the survey packets (four questionnaires); the PISQ-IR was completed by 197 (92 %), and the validation analysis was restricted to these women. According to the answer of the PISQ-IR core-branching item 1, 66 women (33.5 %) considered themselves NSA and 131 (66.5 %) SA. The clinical characteristics of the study population are shown in Table 1. SA women were on average 7 years younger, but otherwise, there were no significant differences between the two groups. Overall, the majority of women (70 %) were postmenopausal, 50 % were diagnosed with symptomatic POP, 74 % with UI, and 4 % with AI. Results of the SF-36 and KHQ subscales (Table 1) indicate that SA women had better general and condition-specific QoL than NSA women. However, differences did not reach statistical significance, except for the two KHQ subscales “role limitations” and “sleep and energy disturbances.”

**Table 1:** Clinical characteristics and quality of life (QoL) questionnaire results of the study population

	Sexually inactive	Sexually active	Total
	*N* = 66	*N* = 131	*N* = 197
Age (mean ± SD)	62 (12)*	55 (12)*	57 (12)
BMI (mean ± SD)	27.5 ( 5.5)	26.8 (4.7)	27.0 (5.0)
Parity (mean ± SD)	2.0 (1.1)	2.3 (1.3)	2.2 (1.2)
Postmenopausal	82 %	62 %	70 %
Past surgical history	*N* = 64	*N* = 118	*N* = 182
Hysterectomy	30 %	26 %	28 %
Prior prolapse surgery	9 %	15 %	14 %
Prior incontinence surgery	22 %	13 %	16 %
Clinical diagnosis	*N* = 63	*N* = 118	*N* = 181
Symptomatic POP	46 %	52 %	50 %
Stress UI	13 %	30 %	24 %
Urge UI	33 %	13 %	20 %
Mixed UI	32 %	30 %	30 %
Anal Incontinence	3 %	5 %	4 %
POP-Q stage	*N* = 44	*N* = 59	*N* = 103
Stage 0 and I	34 %	36 %	35 %
Stage II	46 %	39 %	42 %
Stage III and IV	21 %	25 %	23 %
Oxford Grading Scale	*N* = 58	*N* = 106	*N* = 164
No contraction/flicker	10 %	15 %	13 %
Weak	55 %	39 %	46 %
Moderate	26 %	26 %	26 %
Good/strong	9 %	21 %	16 %
Pelvic floor muscles	*N* = 58	*N* = 105	*N* = 163
Normal	72 %	65 %	68 %
Overactive	2 %	2 %	2 %
Underactive	22 %	30 %	27 %
Nonfunctioning	3 %	4 %	4 %
SF-36 scales^a^	*N* = 65	*N* = 123	*N* = 188
Physical functioning	66.8 (24.5)	70.7 (23.6)	69.3(23.9)
Role physical	58.3 (42.4)	66.6 (39.9)	63.8 (40.8)
Bodily pain	64.9 (30.2)	68.8 (28.3)	67.5 (28.9)
General health perception	59.6 (19.8)	63.6 (19.5)	62.2 (19.6)
Vitality	48.8 (23.5)	54.7 (19.0)	52.6 (20.8)
Social functioning	72.3 (27.8)	79.0 (23.7)	76.7 (25.3)
Role-emotional	63.7 (45.9)	72.7 (42.5)	69.8 (43.7)
Mental health	65.3 (22.1)	66.6 (20.2)	66.2 (20.8)
KHQ scales^b^	*N* = 50	*N* = 91	*N* = 141
General health perception	40.5 (20.1)	39.0 (23.6)	39.5 (22.4)
Incontinence impact	73.3 (30.1)	64.4 (29.9)	67.6 (30.2)
Role limitations	64.2 (31.5)*	52.8 (31.9)*	56.8 (32.1)
Physical limitations	61.6 (34.1)	50.5 (30.3)	54.4 (31.9)
Social limitations	30.8 (30.1)	21.4 (24.9)	24.7 (27.1)
Personal relationship	46.2 (36.1)	29.3 (31.1)	31.6 (32.1)
Emotional problems	45.4 (31.4)	42.5 (30.2)	43.5 (30.6)
Sleep and energy disturbances	50.4 (25.9)*	31.4 (27.9)*	38.1 (28.6)
Symptom severity	69.3 (22.0)	63.9 (27.4)	65.8 (25.7)
Overactive bladder	47.0 (22.2)	45.7 (29.2)	46.1 (26.9)

Data is given in percent or mean (standard deviation)

* p < .05;

^a^ Higher scores indicate better quality of life

^b^ Higher scores indicate worse quality of life

The PISQ-IR results were analyzed separately for SA and NSA women, as recommended in the original publication [6]. Item nonresponse rates ranged from 9 % to 55 % in the NSA group (median 30 %; 20/66) and from 2 % to 40 % in the SA group (median 7 %; 9/131). Table 2 shows mean values, internal consistency reliability, and floor and ceiling effects of the PISQ-IR subscales. Cronbach’s alpha coefficients ranged from 0.64 to 0.94, with two subscales (NSA- CS, and SA-D) being below the acceptable level of 0.7; all other scales indicated a good level of internal consistency.

**Table 2:** Scale parameters of the Pelvic Organ Prolapse/Incontinence Sexual Questionnaire–International Urogynecology Association Revised (PISQ-IR)

Scale	No. of items	Mean ± SD	Cronbach’s alpha	Floor	Ceiling
NSA (N = 66)					
Condition-specific (NSA-CS)	3	32.87 ± 30.64	0.64	28 %	6 %
Partner-related (NSA-PR)	2	75.99 ± 30.68	n.a.	5 %	51 %
Global quality (NSA-GQ)	4	38.23 ± 33.97	0.94	32 %	6 %
Condition impact (NSA-CI)	3	34.61 ± 34.21	0.87	40 %	4 %
SA (N = 131)					
Arousal/orgasm (SA-AO)	3	57.78 ± 22.15	0.81	2 %	2 %
Partner-related (SA-PR)	3	75.07 ± 20.28	0.80	1 %	20 %
Condition-specific (SA-CS)	3	78.17 ± 23.02	0.70	1 %	29 %
Global quality (SA-GQ)	4	67.67 ± 24.77	0.91	3 %	18 %
Condition impact (SA-CI)	4	67.93 ± 29.60	0.87	1 %	30 %
Desire (SA-D)	3	49.05 ± 18.77	0.69	4 %	2 %

Mean scores are transformed to a 0–100 range. In the NSA group, a higher value indicates a greater negative impact. For SA women, a higher value indicates less impact and better sexual function

NSA nonsexually active, SA sexually active, * n.a.* not assessed because <3 items

Ceiling effects were seen for the NSA-PR subscale, with 51 % of women reporting the worst possible score. This indicates that having no partner and having no interest were common reasons for sexual inactivity. Floor effects were found in the NSA-CI, indicating that for 40 % of NSA women, PFD had no impact. Moderate ceiling effects were seen in the subscales SA-CI and SA-CS, where 29 and 30 % of women, respectively, reported the best possible score. This indicates that in those women urogynecological problems had no impact on their sexuality. Table 3 shows the analysis of criterion validity for NSA women. Significant correlations in the anticipated directions were observed between the subscales NSA-CS and NSA-CI and most SF-36 and KHQ subscales. Correlations ranged between 0.34 and 0.93 and were strongest for the SF-36 domain “emotional-role functioning” and the KHQ scale “personal relationship.” There were no significant correlations with clinical measures, except between body mass index (BMI) and NSA-PR.

**Table 3:** Sexually inactive (NSA) Pelvic Organ Prolapse/Incontinence Sexual Questionnaire–International Urogynecology Association Revised (PISQ-IR) scale correlations with 36-Item Short Form Health Survey (SF-36), Kings Health Questionnaire (KHQ), and clinical measures

	Dimension: NSA		Dimension: quality and satisfaction	
Scale	Not sexually active- condition specific	Not sexually active- partner related	Not sexually active-global quality	Not sexually active-condition impact
	NSA-CS	NSA-PR	NSA-GQ	NSA-CI
SF-36 scales ^a^				
Physical functioning	−0.55**	−0.01	−0.17	−0.20
Role-physical	−0.45*	0.11	−0.05	−0.17
Bodily pain	−0.40*	0.17	0.04	−0.27
General health perception	−0.54**	0.07	−0.10	−0.22
Vitality	−0.41*	0.21	−0.16	−0.19
Social functioning	−0.45**	0.03	−0.18	−0.26
Role-emotional	−0.65**	0.13	−0.12	−0.34*
Mental health	−0.44**	0.09	−0.18	−0.26
KHQ scales ^b^				
General health perception	0.54**	−0.11	−0.06	0.10
Incontinence impact	0.49**	−0.11	−0.17	0.53**
Role limitations	0.58**	−0.01	−0.34*	0.28
Physical limitations	0.46*	0.04	−0.30	0.26
Social limitations	0.55**	0.00	−0.25	0.29
Personal relationship	0.86*	−0.21	0.49	0.93**
Emotional problems	0.54**	−0.04	−0.11	0.28
Sleep and energy disturbances	0.45*	0.21	−0.18	0.20
Symptom severity	0.59**	−0.06	0.06	0.62**
Overactive bladder	0.31	0.28	−0.26	−0.08
Clinical measures				
BMI	−0.11	−0.40**	0.25	0.24
Pelvic tone (0 = normal, 1 = not normal)	0.08	0.14	−0.12	−0.15
POP-Q	0.09	0.29	−0.13	0.03
Oxford Grading Scale	0.01	−0.13	0.06	0.11

For POP-Q and the Oxford Grading Scale, Spearman’s rank-sum correlations were computed; all other correlations assessed with Pearson correlations (values >0.50 in bold)

*BMI* body mass index,* POP-Q* Pelvic Organ Prolapse Quantification system,* QoL* quality of life

* p < .05, ** *p* < .005.

^a^ Higher scores indicate better QoL

^b^ KHQ analysis limited to women with urinary incontinence; higher scores indicate worse QoL

Criterion validity analysis for SA women is shown in Table 4. Moderate correlations in the anticipated direction were seen between PISQ-IR subscales, especially SA-OA, SA-CS, and SA-CI, and most SF-36 and KHQ subscales, indicating that both general and condition-specific QoL are positively related to better sexual functioning. Moderate to high correlations were observed between corresponding scales of the FSFI and PISQ-IR—i.e., desire, arousal, orgasm, satisfaction/global quality—confirming good criterion validity. High pelvic floor muscle contractility, i.e. Oxford Grading Scale, was positively correlated with SA-AO. A higher POP-Q stage and lower muscle contractility were correlated with worse SA-CI.

**Table 4:** Sexually active- Pelvic Organ Prolapse/Incontinence Sexual Questionnaire–International Urogynecology Association Revised (PISQ-IR) scale correlations with 36-Item Short Form Health Survey (SF-36), Kings Health Questionnaire (KHQ), Female Sexual Function Index (FSFI), and clinical measures

	Dimension: sexual response			Dimension: quality, satisfaction, desire		
	Sexually active-arousal, orgasm	Sexually active-partner-related	Sexually active-condition-specific	Sexually active-global quality	Sexually active-condition impact	Sexually active-desire
	SA-AO	SA-PR	SA-CS	SA-GQ	SA-CI	SA-D
SF-36 scales ^a^						
Physical functioning	0.47**	0.10	0.37**	0.38**	0.38**	0.18
Role physical	0.35**	0.09	0.30**	0.27**	0.28**	0.07
Bodily pain	0.35**	0.18	0.41**	0.36**	0.30**	0.12
General health perception	0.29**	0.30**	0.39**	0.25**	0.20*	0.18
Vitality	0.22*	0.14	0.41**	0.31**	0.22*	0.19*
Social functioning	0.24**	0.17	0.49**	0.27**	0.30**	0.07
Role-emotional	0.26**	0.09	0.36**	0.14	0.20*	0.11
Mental health	0.22*	0.33**	0.48**	0.32**	0.28**	0.19*
KHQ scales ^b^						
General health perception	−0.34**	−0.24*	−0.50**	−0.33**	−0.30**	−0.30**
Incontinence impact	−0.19	−0.21	−0.33**	−0.34**	−0.36**	−0.08
Role limitations	−0.26*	−0.12	−0.32**	−0.28*	−0.38**	−0.03
Physical limitations	−0.13	−0.09	−0.23*	−0.16	−0.28**	0.08
Social limitations	−0.19	−0.19	−0.46**	−0.13	−0.37**	0.09
Personal relationship	−0.44**	−0.24*	−0.52**	−0.39**	−0.62**	−0.01
Emotional problems	−0.27*	−0.27*	−0.37**	−0.26*	−0.41**	0.02
Sleep and energy disturbances	−0.23*	−0.05	−0.32**	−0.17	−0.34**	0.09
Symptom severity	−0.22*	−0.15	−0.35**	−0.20	−0.33**	0.08
Overactive bladder	−0.15	0.00	0.00	−0.06	−0.03	0.08
FSFI scales ^c^						
Desire	0.42**	0.31**	0.20	0.42**	0.02	0.75**
Arousal	0.61**	0.34**	0.37**	0.44**	0.30**	0.51**
Lubrication	0.46**	0.29**	0.23*	0.35**	0.22*	0.43**
Orgasm	0.61**	0.31**	0.33**	0.45**	0.33**	0.34**
Satisfaction	0.32**	0.38**	0.40**	0.56**	0.34**	0.33**
Pain	0.20	0.19	0.38**	0.42**	0.48**	0.25*
Total score	0.51**	0.35**	0.35**	0.51**	0.34**	0.51**
Clinical measures						
BMI	−0.11	−0.05	−0.10	−0.03	0.00	−0.01
Pelvic floor tone	−0.10	0.03	−0.09	−0.08	−0.11	−0.04
POP-Q	−0.13	−0.00	0.26	0.04	−0.34**	−0.06
Oxford Grading Scale	0.26**	−0.04	0.05	0.09	0.20*	0.13

For POP-Q and the Oxford Grading Scale, Spearman’s rank-sum correlations were computed; all other correlations were assessed with Pearson correlations (values >0.50 in bold)

*BMI* body mass index,* POP-Q* Pelvic Organ Prolapse Quantification system

* p < .05, ** *p* < .005.

^a^ Higher scores indicate better QoL

^b^ KHQ analysis limited to women with urinary incontinence only, higher scores indicate worse QoL

^c^ Higher scores indicate better sexual function

## Discussion

The PISQ-IR is a valuable tool for assessing sexual function in women with PFD, both for clinical practice and for research. The present German translation and clinical validation demonstrates sound psychometric properties and will allow its use in the German-speaking urogynecological population. The qualitative linguistic validation showed a high level of acceptance, and only a few adaptations were necessary. However, during the clinical validation process, a substantial nonresponse rate, especially in the NSA group, was observed for selected single items. This seemed to be due to the varying answering format of these items, and as a consequence, the format was adapted. The high number of missing values may also be explained by the fact that four different questionnaires were used: the PISQ-IR, FSFI, SF-36, and KHQ. However, their combined use allowed us to examine the relationship between various general and condition-specific QoL domains and sexual function in NSA and SA women. Several strong correlations between related subscales were observed, confirming the good criterion validity of the newly translated PISQ-IR. Internal validity was acceptable, with Cronbach’s alpha values >0.70, except for NSA-CS and SA-D. Overall, scale correlations and internal validity were comparable with the original PISQ-IR validation [6].

A strength of the study was the sufficiently large sample size and correlation with clinical parameters, i.e., pelvic muscular function. One advantage of the PISQ-IR is the possibility to evaluate SA as well as NSA women. While in our study population having no partner or having a lack of interest was the most common reason for sexual inactivity; only 40 % reported that PFD had no negative impact at all. Similarly, in the SA group, more than two thirds indicated that urogynecological symptoms at least somehow negatively effected their sexuality. This confirms previous studies, which found that POP and UI commonly impact on women’s sexuality and are likely reasons for sexual inactivity [3, 10–12].

Our results are limited by the fact that we did not evaluate test–retest reliability and scale responsiveness, which were established in the original publication. The relatively high floor and ceiling effects in the NSA group imply that some actual variation in patient data may not be reflected. Only a few women with AI were involved in the validation process, and future research will need to test the validity of the German PISQ-IR in this population.

## Conclusion

This study confirms that sexual function is commonly affected by urogynecological symptoms and should be assessed in women with PFD. The German PISQ-IR is a reliable and valid tool for use in a German-speaking urogynecological population. Future use of this questionnaire will be important to assess the effect of the adapted format on missing values and ceiling effects. Future prospective clinical trials should also confirm the stability and establish the clinical responsiveness of the German PISQ-IR.

## Appendix 1

### PISQ-R Fragebogen

Erkrankungen des weiblichen Genitals und der Harnblase können alle Lebensbereiche betroffener Frauen beeinflussen- auch die Sexualität. Wir sind daran interessiert zu erfahren, wie sich die Beschwerden auswirken, bzw. was durch Therapien verbessert werden kann. Wir würden Sie daher bitten uns einige Fragen über Ihr derzeitiges Sexualleben zu beantworten - alle Antworten werden natürlich vertraulich behandelt!

F1 Was im Folgenden beschreibt Sie am besten:

**Table:**

Überhaupt nicht sexuell aktiv	1 □ ➔	Gehen Sie zu F2 – Abschnitt nicht sexuell aktiv
Sexuell aktiv mit oder ohne Partner mit oder ohne Geschlechtsverkehr	2 □ ➔	Gehen Sie zu F7 – Abschnitt sexuell aktiv

### Abschnitt sexuell nicht aktiv

F2 Im Folgenden sind Gründe angeführt, warum Sie nicht sexuell aktiv sind. Geben Sie bitte an inwieweit die Gründe für Sie zutreffen.

**Table:**

	Stimme sehr zu	Stimme etwas zu	Stimme Eher nicht zu	Stimme überhaupt nicht zu
a Kein Partner	□^1^	□^2^	□^3^	□^4^
b Kein Interesse	□^1^	□^2^	□^3^	□^4^
c Wegen Harn- oder Stuhlverlust oder wegen Senkung	□^1^	□^2^	□^3^	□^4^
d Wegen meiner anderen Gesundheits-probleme	□^1^	□^2^	□^3^	□^4^
e Schmerzen	□^1^	□^2^	□^3^	□^4^

F3 Vermeiden Sie sexuelle Aktivität auch aus Angst vor Harn oder Stuhlverlust bzw. aufgrund eines Senkungsgefühls in der Scheide?

1 □ Überhaupt nicht

2 □ Ein bisschen

3 □ Etwas

4 □ Sehr

F4 Wie sehr stimmen Sie den folgenden Aussagen zu (nicht zu)

**Table:**

	Stimme sehr zu	Stimme ziemlich zu	Stimme Mäßig zu	S stimme eher nicht zu	Stimme überhaupt nicht zu
a. Ich bin mit meinem Sexualleben zufrieden	□^1^	□^2^	□^3^	□^4^	□^5^
b. Ich empfinde mein Sexualleben passend für mein Alter und meine Lebensumstände	□^1^	□^2^	□^3^	□^4^	□^5^

F5 Wie sehr stimmen Sie den folgenden Aussagen zu (nicht zu)

**Table:**

	Stimme sehr zu	Stimme etwas zu	Stimme Eher nicht zu	Stimme überhaupt nicht zu
a. Mein Sexualleben frustriert mich	□^1^	□^2^	□^3^	□^4^
b. Ich fühle mich wegen des Harnverlustes und/oder meiner Senkung sexuell benachteiligt	□^1^	□^2^	□^3^	□^4^
c. Ich ärgere mich, weil der Harnverlust und/oder die Senkung mein Sexualleben beeinflussen.	□^1^	□^2^	□^3^	□^4^

F6 Wie sehr stört es Sie, dass Sie nicht sexuell aktiv sind?

1 □ Überhaupt nicht

2 □ Ein bisschen

3 □ Etwas

4 □ Sehr

### Ende des Abschnittes Sexuell nicht aktiv

#### Abschnitt sexuell aktiv

F7 Wie oft fühlen Sie sich während sexueller Aktivität erregt (körperlich oder gefühlsmäßig)?

1 □ Nie

2 □ Selten

3 □ Manchmal

4 □ Meistens

5 □ Fast immer

F8 Wenn Sie sexuell aktiv sind, wie oft haben Sie folgende Gefühle?

**Table:**

	nie	selten	manchmal	oft	Fast immer
a. Befriedigung	□^1^	□^2^	□^3^	□^4^	□^5^
c. Scham	□^1^	□^2^	□^3^	□^4^	□^5^
d. Angst	□^1^	□^2^	□^3^	□^4^	□^5^

F9 Wie oft verlieren Sie Harn und/oder Stuhl bei irgendeiner sexuellen Aktivität?

1 □ Nie

2 □ Selten

3 □ Manchmal

4 □ Oft

5 □ Fast immer

F10 Verglichen mit sexuellen Höhepunkten, die Sie in der Vergangenheit hatten, wie intensiv sind Ihre sexuellen Höhepunkte jetzt?

1 □ Viel weniger intensiv

2 □ Weniger intensiv

3 □ Gleich intensiv

4 □ Intensiver

5 □ Viel intensiver

F11 Wie oft haben Sie beim Geschlechtsverkehr Schmerzen? (Wenn Sie keinen Geschlechtsverkehr haben kreuzen Sie dieses Kästchen □ an und gehen Sie zur nächsten Frage weiter)

1 □ Nie

2 □ Selten

3 □ Manchmal

4 □ Oft

5 □ Immer

F12 Haben Sie einen Sexualpartner/eine Sexualpartnerin?

1 □ Ja ➔ Weiter mit F 13

2 □ Nein ➔ Weiter mit F15

F13 Wie oft hat Ihr Partner/Ihre Partnerin ein Problem (Mangel an Erregung, Bedürfnis, Erektion, etc.), das Ihre sexuelle Aktivität einschränkt?

1 □ Immer

2 □ Meistens

3 □ Manchmal

4 □ Fast nie/selten

F14 Würden Sie sagen, dass Ihr Partner/Ihre Partnerin im Allgemeinen einen positiven oder negativen Einfluss auf folgenden Bereich hat?

**Table:**

	sehr Positiv	Eher Positiv	eher Negativ	sehr Negativ
a. Auf Ihre sexuelle Lust	□^1^	□^2^	□^3^	□^4^
b. Auf die Häufigkeit Ihrer sexuellen Aktivität	□^1^	□^2^	□^3^	□^4^

F15 Wenn Sie sexuell aktiv sind, wie oft haben Sie das Gefühl, dass Sie mehr möchten?

1 □ Nie

2 □ Selten

3 □ Manchmal

4 □ Oft

5 □ Immer

F16 Wie häufig haben Sie ein sexuelles Verlangen (das kann Wunsch nach Geschlechtsverkehr, sexuelle Gedanken oder Fantasien etc. einschließen)?

1 □ Täglich

2 □ Wöchentlich

3 □ Monatlich

4 □ Weniger als einmal im Monat

5 □ Nie

F17 Wie würden Sie den Grad Ihres sexuellen Bedürfnisses oder Interesses beurteilen?

1 □ Sehr hoch

2 □ Hoch

3 □ Mäßig

4 □ Niedrig

5 □ Sehr niedrig oder gar nicht vorhanden

F18 Vermeiden Sie sexuelle Aktivität aus Angst vor Harn oder Stuhlverlust bzw. aufgrund eines Senkungsgefühls in der Scheide?

1 □ Überhaupt nicht

2 □ Wenig

3 □ Etwas

4 □ Sehr

F 19 Wie sehr stimmen Sie den folgenden Aussagen zu (nicht zu)?

**Table:**

	Stimme sehr zu	Stimme ziemlich zu	Stimme Mäßig zu	S stimme eher nicht zu	Stimme überhaupt nicht zu
a. Ich bin mit meinem Sexualleben zufrieden	□^1^	□^2^	□^3^	□^4^	□^5^
b. Ich empfinde mein Sexualleben passend für mein Alter und meine Lebensumstände	□^1^	□^2^	□^3^	□^4^	□^5^
c. Ich fühle mich in meinem Sexualleben sicher und zuversichtlich	□^1^	□^2^	□^3^	□^4^	□^5^

F20 Wie sehr stimmen Sie den folgenden Aussagen zu (nicht zu)?

**Table:**

	Stimme sehr zu	Stimme etwas zu	Stimme EHER nicht zu	Stimme überhaupt nicht zu
a. Mein Sexualleben frustriert mich	□^1^	□^2^	□^3^	□^4^
b. Ich fühle mich wegen des Harnverlustes und/oder meiner Senkung sexuell benachteiligt	□^1^	□^2^	□^3^	□^4^
c. Mein Sexualleben ist mir unangenehm	□^1^	□^2^	□^3^	□^4^
d. Ich ärgere mich, weil der Harnverlust und/oder die Senkung mein Sexualleben beeinflussen	□^1^	□^2^	□^3^	□^4^

Herzlichen Dank!
